# Systematic review of first-in-human and early phase clinical trials for surgically implantable biological mitral valve substitutes

**DOI:** 10.1186/s13019-023-02464-2

**Published:** 2023-11-30

**Authors:** Faizus Sazzad, Ying Kiat Tan, Li Xuan Beverly Chan, Irwan Shah Bin Mohd Moideen, Abdulrahman El Gohary, John C. Stevens, K. R. Ramanathan, Theo Kofidis

**Affiliations:** 1 Department of Surgery, Yong Loo Lin School of Medicine, Centre for Translational Medicine, National University of Singapore, MD6, 14 Medical Drive, Level-8 (South), Singapore, 117599 Singapore; 2 Department of Cardiac, Thoracic and Vascular Surgery, National University Heart Centre, National University Hospital, Singapore, Singapore

**Keywords:** Mitral valve, Heart valve prosthesis, Bioprosthesis, First-in-human, Clinical trial, Systematic review

## Abstract

**Background:**

The aim of this review was the creation of uniform protocols to carry out and disclose First-In-Human and preliminary clinical trials of biological mitral valve replacement. The need for consistent methodology in these early trials was highlighted by the observation of significant variability in the methods and protocols used across different research.

**Methods:**

An extensive search through six major databases was carried out to retrieve First-In-Human (FIH) clinical studies evaluating surgically implanted bio-prostheses in the mitral position.

**Results:**

Following the PRISMA guideline, a systematic search identified 2082 published articles until March 2023. After removing duplicates (189), 1862 citations were screened, resulting in 22 eligible studies with 3332 patients for analysis. The mitral valve prostheses in these studies ranged from 21 to 37 mm, with the 29 mm size being most prevalent. Patient numbers varied, with the FIH subgroup including 31 patients and the older subgroup including 163 patients. Average study durations differed: the older subgroup lasted 4.57 years, the FIH subgroup 2.85 years, and the early phase studies spanned 8.05 years on average.

**Conclusion:**

FIH clinical report is essential to assess the significance of clinical data required for a “de novo” surgical implant. In addition, understanding the performance of the device, and recognizing the difficulties associated with the innovation constitute important lessons. These insights could be beneficial for the development of bioprosthetic heart valves and formulating a protocol for an FIH clinical trial.

**Supplementary Information:**

The online version contains supplementary material available at 10.1186/s13019-023-02464-2.

## Introduction

Clinical results for individuals with valvular heart disease improve when a functional valve substitute (such as surgical implants or biological valve prostheses) is used [1]. Although the Hufnagel valve’s initial design was simple, throughout the course of the following 40 years, other manufacturers created and tested a variety of bioprosthetic valves of diverse designs, from Hancock porcine valves to Carpentier Edwards Valves [2]. The leverage requirement for successful device testing in the clinical setting was demonstrated by lessons learned from the history of cardiac device development. The First-In-Human (FIH) and initial clinical trial have been the main barriers to the development of cardiac devices thus far.

According to the general definition of functional valve substitutes, there are two categories of surgical valves that can be used for treatment: (1) mechanical valves, which are composed of different metals, and polymers, and (2) carbon and bioprosthetic valves, which are made of biological tissue and/or Xenoprostheses [3]. A bio-prosthetic valve is currently chosen by 60% to 70% of patients, and this is becoming more popular. Surgical tissue valves are often bovine pericardial valves, which require less anticoagulation for a short time [4].

During the development of heart valves, many studies reported the clinical outcomes and long-term follow-ups at various stages of their respective clinical trials. It is important to note that reporting procedures differed significantly between studies. There was no standard procedure for reporting clinical results for biological valve replacements, particularly for the mitral position. This is in contrast with the majority of other interventional research, such as clinical trials with established protocols to evaluate the success of the intervention. Thus, it is necessary to standardize the reporting of results for all heart valve replacement studies.

Last but not least, the majority of research evaluating new heart valves for the different anatomical positions [5] or studies comparing the performance of mechanical or biological prostheses [6] make up the present scientific literature on heart valves. However, there is not enough research on how to carry out FIH clinical trials for mitral valve replacement. FIH and initial clinical trials are critical translational steps to test the safety of any new cardiac device. The safety of participants is the most important factor when moving forward with FIH and early clinical trials in human beings. Even though there have been several FIH studies on mitral valve substitutes, there is no agreement on the outcomes that should be reported, leading to trials with wildly disparate study designs.

There isn’t enough literature on how to perform First-In-Human clinical trials for bioprosthetic mitral valve replacement or on the ideal protocol to use when evaluating mitral valve bio-prostheses. Functional valve substitutes are subject to regulatory authority approval processes for De Novo devices and Class III with or without predicate devices.. The results sought from clinical studies are concentrated on safety, efficacy, and less rigorous clinical evidence, even though the approval process from various authorities has its distinctions and commonalities [7]. In order to offer an acceptable study design that will strategically aid to the development of a novel mitral valve, this review was undertaken to evaluate the study protocol of past initial clinical trials and FIH studies.

## Material and methods

We conducted the systematic review following the Preferred Reporting Items for Systematic Reviews and Meta-analyses for Systematic Review (PRISMA) guidelines [8]. In addition, we searched electronic databases on Medline (via PubMed), Scopus, Embase, ScienceDirect, Web of Science, and Cochrane database records from the date of inception to March 10, 2023. To identify First-In-Human and initial clinical trials that evaluated any new mitral valve bioprosthesis that was surgically implanted in adult patients, a repetitive and exhaustive combination of the following search terms including ‘Medical Subject Headings’ (MeSH) were used: “heart valve prosthesis,” “bioprosthesis,” “mitral valve.” This review was restricted to articles published in English. This study protocol was registered with PROSPERO (CRD42020223176) before the commencement of the study.

### Study groups

We categorized our included studies into a total of three subcategories—older generation and newer generation group, with the newer generation group further divided into FIH and early phase clinical trials group. Studies from earlier generation were those published before 1990. Newer generation FIH and early phase clinical trials were studies published after 1990.

### Enrolment criteria

The main inclusion criterion for a publication to be considered was its assessment of mitral valve bio-prostheses in First-In-Human or early clinical trials. Studies discussing both mitral and aortic valve replacement were also considered.. However, only mitral valve data was extracted from this combination. In addition, only relevant studies related to the search terms were included. Finally, only studies with unambiguous First-In-Human and preliminary clinical evidence for a novel mitral valve bio-prosthesis were considered. Animal studies or those assessing mechanical prostheses were excluded. Studies that employed bioprostheses or xenografts for transcatheter mitral valve replacement were also disregarded. Studies lacking information on the First-In-Human trial design, such as patient demographics, prosthesis type, and clinical trial type, were excluded. Retrospective studies, meta-analyses, systematic reviews, descriptive papers, case reports and series, ideas, editorials, and perspectives were excluded as their design was not FIH or early phase clinical trials. Additionally, studies that did not have an available English translation were also excluded.

### Data abstraction and outcome

For inclusion, three authors (Y.K.; L.C.; F.S.) independently screened and evaluated the study design’s specifics. In order to incorporate all pertinent studies, the titles and abstracts of articles were initially examined. If the authors were unable to verify the study’s applicability for inclusion, a full-text review of the publications was conducted. Next, the demographics of the study population (type of valve used, number of patients, type of clinical trials, length of the study, follow-up time), the reason for surgery, the size of the prosthesis utilized, and the outcomes of interest were independently abstracted by the three authors (Y.K.; L.C.; F.S.) (hemodynamic performance, consequences of morbid events, morbidity).

### Quality of evidence and risk of bias assessment

We utilized GRADEpro to assess the quality of evidence in the included studies (Additional file 1: Table S1), following the guidelines outlined in Chapter 11 of the Cochrane Handbook of Reviews. The articles were evaluated for bias risk and overall evidence quality. Additionally, for the non-randomized studies included, we utilized the ROBINS-I tool (Risk of Bias in Non-randomized Studies-of Interventions) to evaluate the risk of bias. Following the Grade approach, each study was graded based on bias risk, consistency risk, imprecision risk, indirectness risk, and publication bias risk.

In all investigations, both bias risk and indirectness were considered low due to the suitability of the study designs for first-in-human (FIH) and initial clinical trials. As there is no standardized reporting method for FIH trials, inconsistency and imprecision were not deemed serious, even though each FIH study had a unique design and reporting method. This paper seeks to address the lack of standardized reporting in FIH trials.

### Statistical analysis

SPSS, Statistical Package for the Social Sciences, version 27.0, IBM (2020) was used for data analysis. Levene’s test was used to assess the homogeneity of variances among the incidences of various categories. The data were examined using one-way ANOVA if the variances were found to be homogenous, and post-hoc comparisons using the Tukey’s test were done to see if there were any significant differences between the three groupings. If the variances were not homogeneous, Welch ANOVA was used to examine the data, and the Games-Howell Test was used for post hoc comparisons.

## Results

A comprehensive electronic search based on the previously mentioned approach retrieved 3437 articles from PubMed, Embase, and Web of Science. A further 31 publications were found after the reference list of the chosen articles was additionally examined to find pertinent studies. After using EndNote X9 reference management software to eliminate 362 duplicate citations, a total of 3106 citations were chosen for screening.

### Characteristics of included studies

**Fig. 1:**
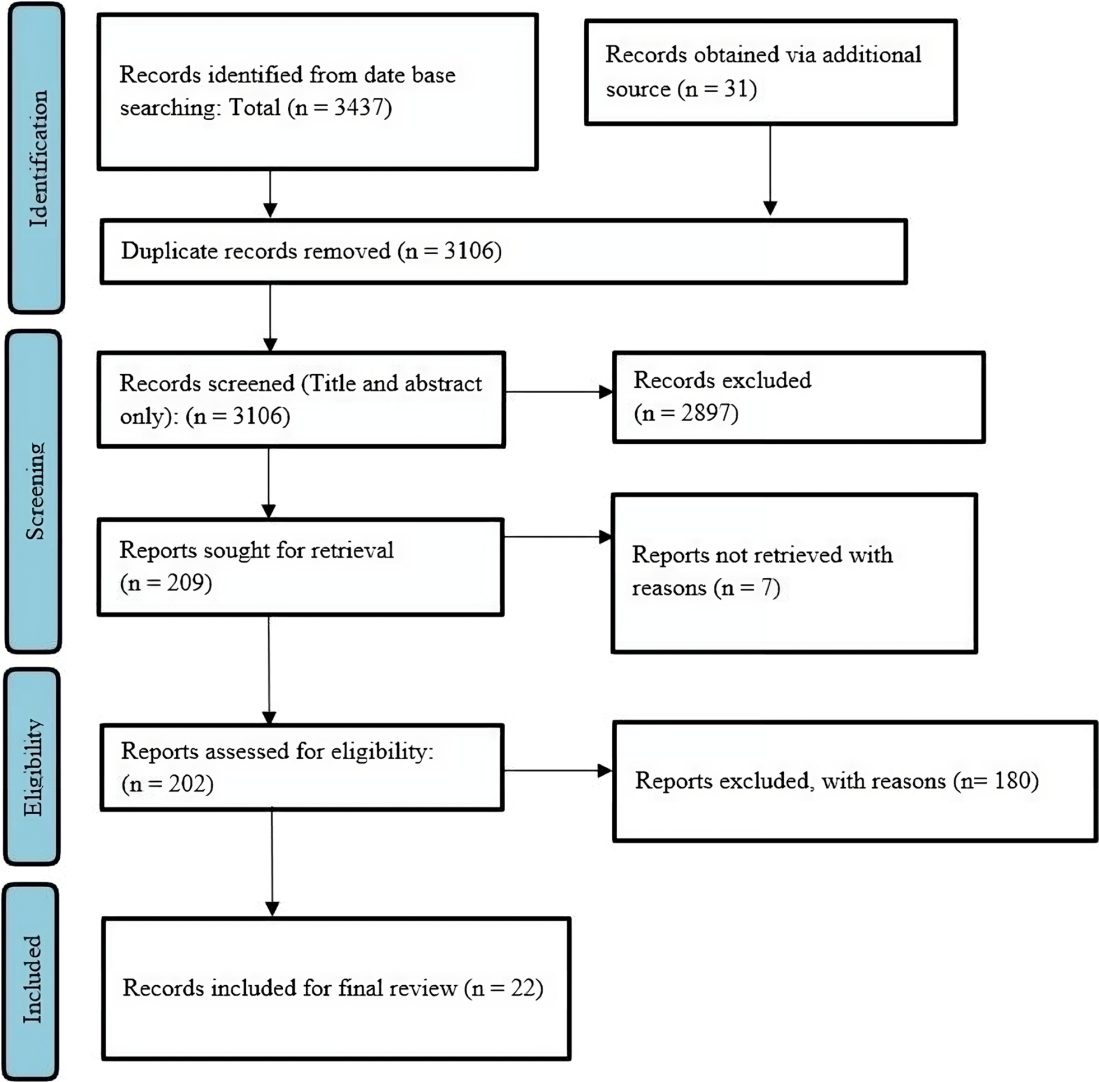
The PRISMA flow diagram shows the identification of the sequential steps in the study selection process, illustrating the identification of 3437 records and the gradual selection leading to the inclusion of the final 22 articles

On the basis of the abstract and title, 2897 irrelevant citations were eliminated. After further assessment of the full text of the remaining citations, 187 were excluded because they did not meet the enrolment criteria. Therefore, based on our search, 22 studies [9–30] with 3332 patients were found to be eligible. Figure 1 shows a Prisma graphic that details the flow of study identification.

**Fig. 2:**
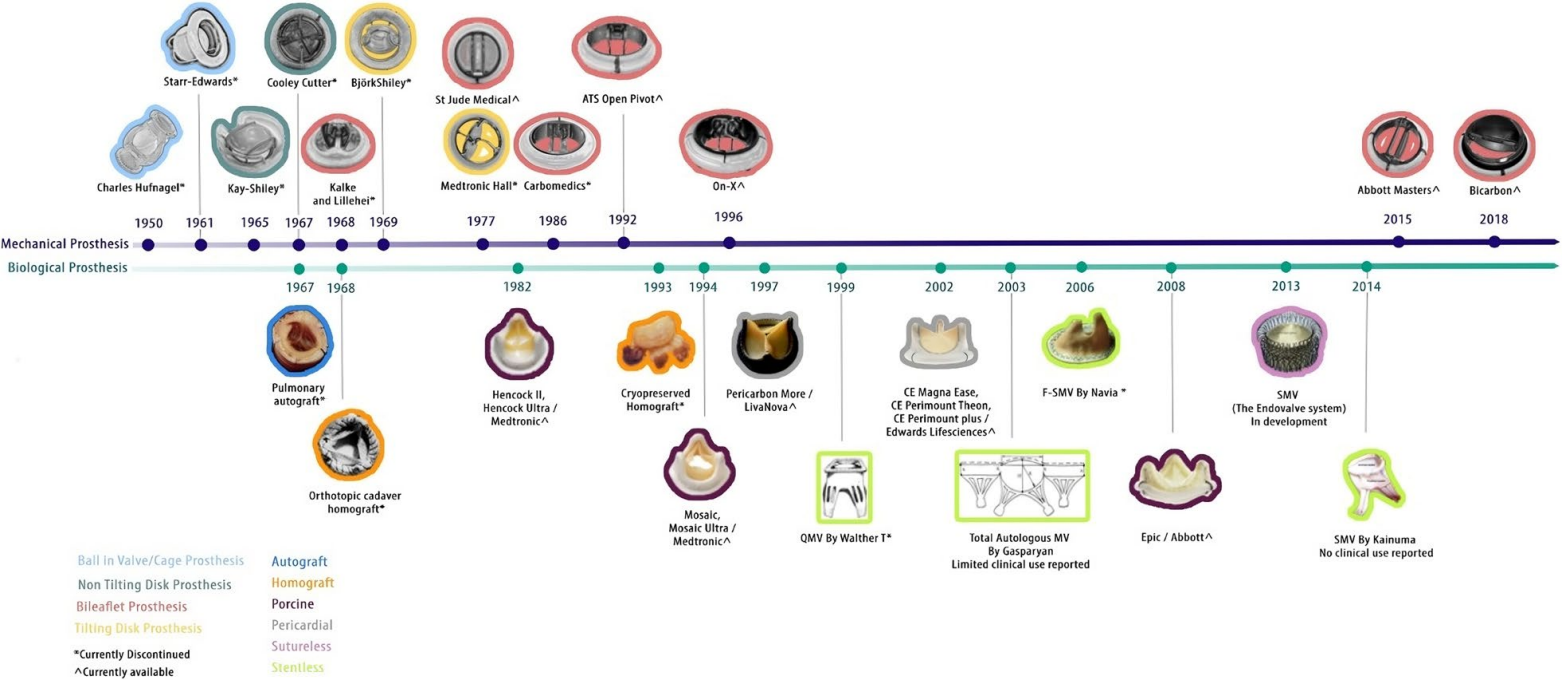
The timeline illustrates the commercialization and commencement of trials for First-In-Human (FIH) and early clinical assessments of heart valve prostheses spanning from 1950 to 2018. The upper section represents the evolution of mechanical heart valve prostheses, while the lower section showcases various biological mitral prostheses. Mechanical prostheses are grouped into four categories: (i) Ball in valve/cage (sky blue), (ii) Non-tilting disk (green), (iii) Tilting disk (yellow), (iv) Bileaflet (red). Biological prostheses are classified into six categories: (i) Autografts (blue), (ii) Homografts (orange), (iii) Porcine (purple), iv) Pericardial (ash), (v) Sutureless (pink), and (vi) Stentless (light green). NB: *indicates currently discontinued, and ^signifies currently available

Figure 2 shows the timeframe for the development of heart valve prosthesis. 10 innovative mitral valve bioprostheses were able to advance to the First-In-Human clinical trial stage and beyond over the course of 50 years.

Our included studies were divided into 3 subcategories, allowing us to compare and contrast the traits and results of each subgroup. One of Carpentier-Edwards, three Medtronic valves, and the Quattro valve had multiple papers reporting on the same valve. The valves were the Carpentier-Edwards 3rd generation supra-annular valve [9, 10], Medtronic Mosaic [19, 23, 27, 30] Medtronic Intact [11, 12, 17], Medtronic Hancock II [14, 24], Quattro Valve [18, 26]. Even though the same valve was reported, the studies were divided into various author-defined groups, with mean values that were representative of that group (older generation, FIH, or early phase clinical trials). It was made sure that the trials featured separate institutes and, as a result, a distinct group of patients for those investigations designated inside the same subgroup.

The older, FIH, and initial clinical trials average duration was 4.57 years, 2.85 years, and 8.05 years, respectively. The average number of patients who underwent mitral valve surgery in the older FIH and initial clinical trials was 163, 31, and 154, respectively. The average follow-up duration of the older FIH and initial studies was 4.85 years, 2.3 years, and 4.21 years respectively (Table 1). As a result, the FIH studies often enroll fewer patients and have shorter study and follow-up, which is consistent with the criteria of Phase I of the clinical trial testing for adverse effects. With the exception of Riess et al. [27] (70%) in all studies, the follow-up completion rate is above 91% [27].

**Table 1:** Characteristics of the included studies

References	Prosthesis used	Duration	Number of patients	Place of study	Follow up range (years)	Follow up duration (years)	Follow up completeness
*Relland *[9]	Carpentier-Edwards 3rd generation supra-annular valve (porcine)	4 years 3 months	145	France	NA	7	NA
*Jamieson *[10]	Carpentier-Edwards 3rd generation supra-annular valve (porcine)	2 years 3 months	259	Canada	NA	NA	98.60%
*Jamieson *[11]	Medtronic Intact (porcine)	11 years	333	Canada	NA	10	95.30%
*Williams *[12]	Medtronic Intact (porcine)	3 years	114	South Africa	NA	1.66	NA
*Loisance *[13]	Sorin Mitroflow (pericardial)	2 years 9 months	52	France	2.9—5.5	3.5	100%
*Bortolotti *[14]	Medtronic Hancock II (porcine)	4 years 2 months	72	Italy	0.6—4.5	2.1 ± 1.2	100%
**Garcia **[15]	Labcor-Santiago (pericardial)	8 months	11	Spain	NA	NA	100%
**Wheatley **[16]	Bioflo (bovine pericardial)	3 years 1 month	31	Scotland	Max:7.47	5.45 ± 1.93	100%
**Vermeulen **[17]	Medtronic Intact (porcine)	2 years 11 months	438	Worldwide	Max: 5	N.A	NA
**Mohr **[18]	Quattro (stentless)	NA	52	Germany	0.08—5.41	0.26 ± 0.13	100%
**Fradet **[19]	Medtronic Mosaic (porcine)	6 years 7 months	366	Worldwide	Max: 6.1	2.5	NA
**Hiremath **[20]	Dafodil (stented, bovine, pericardial)	12 months	30	India	NA	1	93.33%
***Firstenberg ***[21]	Carpentier-Edwards Perimount (pericardial)	1 year 1 month	69	Worldwide	0.30—2.44	1.62 ± 0.36	100%
***Folliguet ***[22]	Sorin Pericarbon (bovine pericardial)	9 years 2 months	39	France	NA	4.9 ± 2.6	NA
***Eichinger ***[23]	Medtronic Mosaic (porcine)	5 years 3 months	100	Europe	0—6.1	2.6	98%
***Masters ***[24]	Medtronic Hancock II (porcine)	9 years 8 months	138	Canada	NA	8.33	97%
***Pomerantz ***[25]	St Jude Medical Biocor (porcine)	17 years 9 months	546	Brazil	NA	4.35	NA
***Frater ***[26]	Quattro (stentless)	7 years 11 months	175	South Africa, Germany, Saudi Arabia	0—7.5	3.4	91%
***Riess ***[27]	Medtronic Mosaic (porcine)	5 years 8 months	47	USA	Max:10	5.4	70%
***Jamieson ***[28]	St Jude Medical Epic (porcine)	4 years	175	USA	NA	NA	94.80%
***Loor ***[29]	Carpentier-Edwards Perimount Magna (bovine pericardial)	4 years	70	USA	0—5.12	1.3	NA
***Celiento ***[30]	Medtronic Mosaic (porcine)	16 years	100	Italy	Max: 17.7	6 ± 4.6	97%

NB: italic: Older FIM; Bold: FIM; Bolditalic: Early Clinical trials; NA = Not applicable

### Preoperative demographics of patients

The average patient age of the older generation, FIH, and initial studies were 55.75, 61.06, and 67.44 years respectively, with the mean age for each study ranging from 39 to 73 years old. Only 2 out of 6 of the older studies reported preoperative NYHA Class of their patients, while all FIH and initials studies did so. All the data were reported as percentages per NYHA Class, except as an average Class in Mohr et al. [18] (Table 2).

**Table 2:** Preoperative demographics of patients

References	Gender	Range of patient age (in years)	Average patient age (in years)	Preoperative NYHA Class				Associated/concomitant procedures
				I	II	III	IV	
*Relland *[9]	NA	NA	NA	NA				NA
*Jamieson *[10]	NA	NA	NA	NA				NA
*Jamieson *[11]	527F, 745 M	9–91	67	NA				NA
*Williams *[12]	100F, 67 M	5–78	39	NA				NA
*Loisance *[13]	82F, 84 M	14–83	58 ± 13	6.02%		69.90%		33.1% (55)
*Bortolotti *[14]	NA	29–76	59 ± 8		5.38%	88.50%	6.15%	18.4% (24)
**Garcia **[15]	32F, 8 M	32–81	65.6			76%		31% (12)
**Wheatley **[16]	25F, 6 M	38–69	57.6 ± 9.9	3.23%	38.70%	54.80%	3.23%	22.6% (7)
**Vermeulen **[17]	945F, 520 M	NA	58.6 ± 14.8		6.90%	60.00%	31.60%	11% (48)
**Mohr **[18]	36F, 16 M	NA	68 ± 8.5	Average 3.1 ± 0.6				
**Fradet **[19]	194F, 172 M	17–84	68			79.20%		42.30%
**Hiremath **[20]	18F, 12 M	18–72	48.57 ± 12.63			96.67%	3.33%	33.3% (10)
***Firstenberg ***[21]	33F, 36 M		72.3 ± 5.5			84%		
***Folliguet ***[22]	68F, 32 M	41–84	69		14.40%	49.50%	32.10%	31% (86)
***Eichinger ***[23]	261F, 300 M			0.00%	19.00%	58.00%	23.00%	64% (64)
***Masters ***[24]	78F, 60 M	38–85	72 ± 0.8	14%	12%	43%	21%	67% (92)
***Pomerantz ***[25]	320F, 226 M				2.20%	55.90%	41.90%	32.7% (179)
***Frater ***[26]	126F, 49 M	12–87	46			62.5%	16.50%	45.7% (80)
***Riess ***[27]	33F, 14 M	41–84	67	0%	36.20%	59.60%	4.30%	
***Jamieson ***[28]	98F, 77 M	44.5–91.4	72.2 ± 8.9			61.70%		
***Loor ***[29]	34F, 36 M	29–88	68	57%		43%		83% (58)
***Celiento ***[30]	36F, 64 M		73 ± 10	6%	9%	61%	24%	41% (41)

NB: italic: Older First-in-man (FIM); bold: FIM; bolditalic: Early Clinical trials; NYHA = New York Heart Association; NA = Not applicable; M = Male, F = Female

### Indications for surgery

**Fig. 3:**
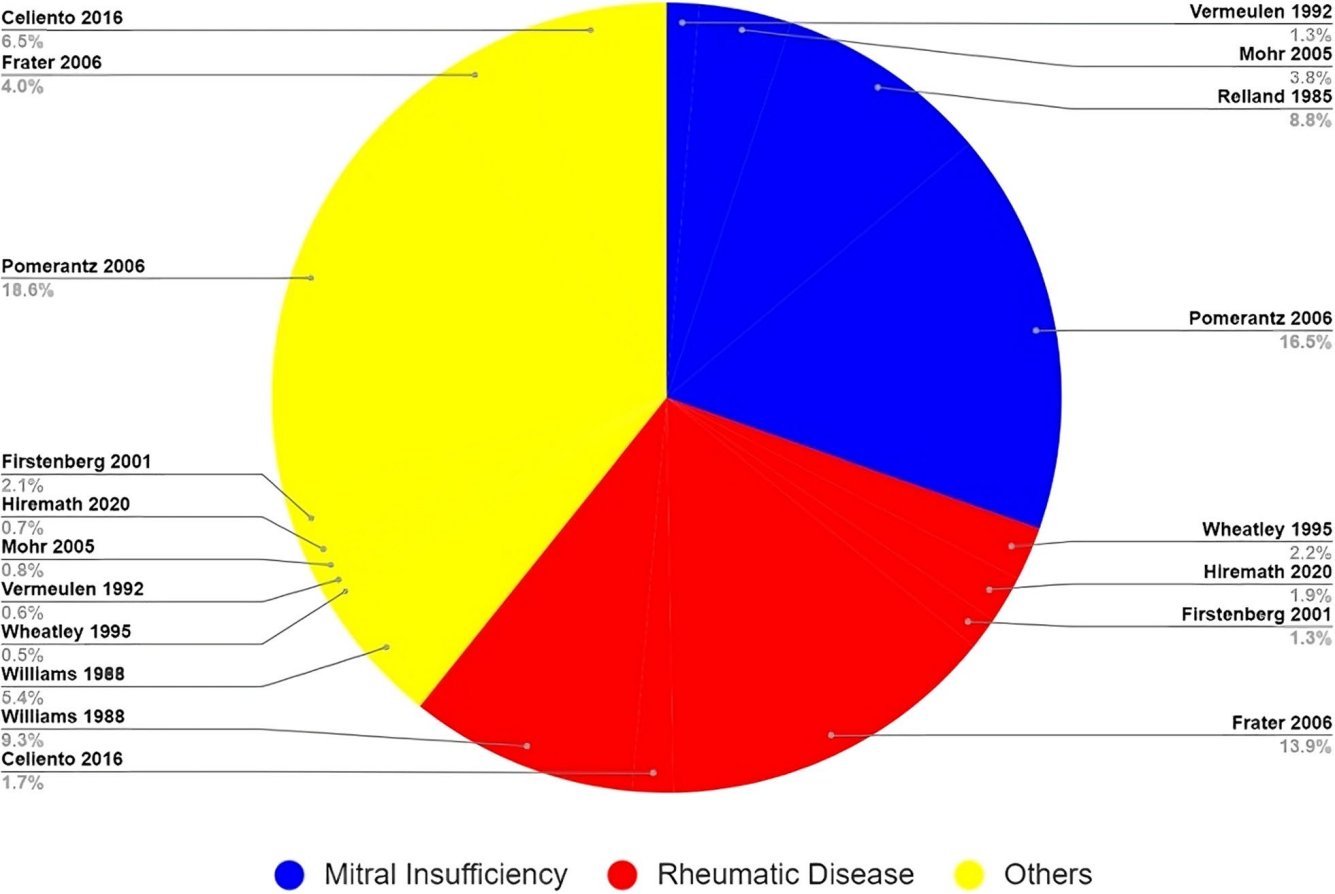
An illustration of the indications for mitral valve replacement surgery using a unique implantable biological mitral valve prosthesis is shown in the pie chart. Rheumatic stenosis and degenerative mitral insufficiency made up the majority of the study’s causes

Data on the indication for surgical mitral valve replacement is shown in Fig. 3, which displays the number of patients undergoing surgery.These are grouped into the following categories: mitral insufficiency, rheumatic disease, endocarditis, congenital/traumatic/ischemic disease, degenerative disease, mixed lesions, and others.

### Mitral valve prosthesis size

**Fig. 4:**
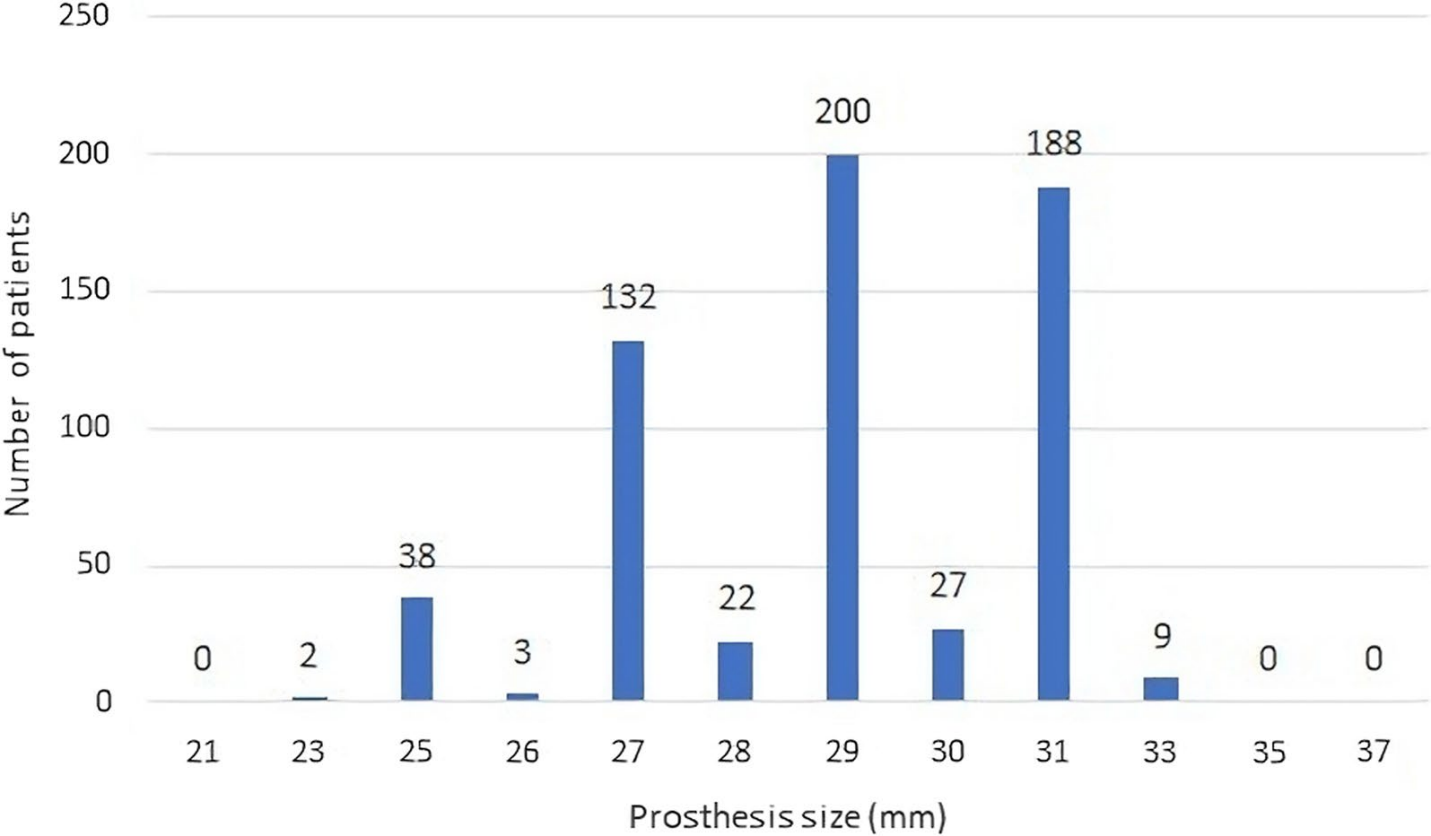
The bar graph shows how biological mitral valve replacement prostheses that have recently been implanted are being used. Insight into the patterns of size preferences in clinical practice is provided by the distribution of various sizes that have been used

Data on prosthesis sizes were present in 12 out of the 22 studies (Additional file 1: Table S2). However, Loisance et al. [13], Garcia et al. [15], Riess et al. [27] were not included in Additional file 1: Table S2 as they only mentioned that valves implanted were of sizes 27 mm and 29 mm without providing data on the exact number of patients that had each valve implanted. Jamieson [28] was also not included as the percentages reported under each prosthesis size did not correlate with the absolute number of patients. Figure 4 shows the number of patients with mitral valve replacement with prosthesis sizes ranging from 21 to 37 mm. A 29 mm size was inserted in 197 patients. The lowest prosthesis size used was 25 mm whilst the highest was 31 mm.

### Hemodynamic profile of mitral valve prosthesis

The most commonly reported haemodynamic data were effective orifice area (EOA), mean diastolic gradient (MDG), peak diastolic gradient (PDG) found in (Additional file 1: Table S3). Nevertheless, only 12 out of 22 studies reported these data. Of these 12 studies, hemodynamic data were most commonly reported in the following time frame after implantation: postoperative (3/12), one year (3/12), and five years (3/12). Instead of directly stating the values for hemodynamic data, some studies [28, 29] used only graphical representation to report hemodynamic data. For example, Wheatley et al. [16] used graphs to accompany numerical values stated in prose form.

Regarding the remaining 10 articles without haemodynamic data, four [11, 13, 17, 26] published their data separately whilst one [14] claimed that it was due to its preliminary nature and another [22] justified the absence of haemodynamic quantification due to its focus on structural valve deterioration and long-term durability.

### Morbidity and consequences of morbidity

According to the Levene’s test of homogeneity of variances (Additional file 1: Table S4A), outcomes such as reoperation, late mortality, thromboembolism, haemorrhage, and endocarditis showed non-uniform variances. Early deaths (*p* = 0.008), SVD (*p*0.001), and periprosthetic leak (*p*0.001) all showed heterogeneity in the test of variance. However, no category exhibiting homogeneous variance showed significantly different results across the three groupings. The three outcomes stated in Additional file 1: Table S4B were examined using Welch ANOVA, and the results showed no statistically significant differences among the three subgroups in any category. One-way ANOVA analysis was performed on the remaining outcomes with homogeneous variances, as shown in Additional file 1: Table S5. Additionally, the subgroup analysis found no notable variations across the groups.

## Discussion

### Characteristics of included studies

The study design from the FIH trial and early clinical investigations varied from generation to generation. The average duration of each study increased from the oldest generation to the newest generation (4.57 years to 8.05 years). A significant variation in the number of patients was also observed between studies from each generation. The average number of patients ranged from 31 patients (FIH subgroup) to 163 patients (older subgroup). Noticeably, 12 out of all 22 included studies had more than 100 patients. This number is noticeably larger than most First-In-Human studies in general, ranging from 20 to 80 patients [7]. This could be explained by the fact that some studies essentially reported on outcomes as the trial progressed from a Phase I to a Phase II/III clinical trial, resulting in many patients observed.

It is also worth noting that there was a significant variation in the follow-up duration, with the average follow-up duration among groups ranging from 2.74 years (FIH subgroup) to 4.852 years (Older group).Follow-up duration ranged from less than one year to more than ten years.. This large variance could again be explained by the fact that some studies reported on outcomes as the trial progressed from a Phase I to Phase III trial, or those different companies made different decisions for the length of follow-up they were interested in. It should be recommended that all First-In-Human studies should have around 20–40 patients, and early phase clinical trials should have around 150–200 patients. The reporting of outcomes at different phases of a clinical trial could also be done in separate studies. The follow-up duration should be a minimum of one year, with a maximum of 2–4 years. These recommendations would ensure that the reporting of outcomes is comparable, allowing researchers to conduct further analysis on the performance of a new mitral valve compared to previous FIH studies that followed the same study design. The geographical distribution of centers where the trials were conducted suggested that most of the studies were conducted in North America (USA, Canada) and Europe (Italy, France, Germany, Spain), with a small minority of studies conducted in South Africa.

### Indication for surgery

The indication for mitral valve replacement surgery remained relatively constant across the three subgroups; the indication for surgery mainly was due to mitral insufficiency or rheumatic heart disease. However, the observed trend was that mitral regurgitation was the indication for surgery in studies conducted in developed countries (France, Germany) whilst rheumatic disease was the main reason in developing countries. This could be explained by the fact that rheumatic heart disease tends to be less prevalent in developed countries [31, 32].

### Hemodynamic profile of mitral valves

Across all studies, there were multiple inconsistencies in the way hemodynamic data were reported. Firstly, some studies reported other types of hemodynamic data besides the Effective Orifice Area (EOA), Peak Diastolic Gradient (PDG), or Mean Diastolic Gradient (MDG), such as Left Ventricular Ejection Fraction instead for instance. Among the studies that reported EOA/PDG/MDG, there was further inconsistency as some studies only reported one out of the three variables or only reported the overall value for the variable that was not specific for each valve size. Even within the 12 studies that reported EOA/PDG/MDG, they reported the data collected at different months, due to the difference in the follow-up periods, for example, 600 days in Firstenberg [21] and 6.9 years in Celiento [30]. Lastly, other studies reported the data in bar charts without any specific numbers, preventing further analysis. To allow a fair comparison between the mitral valves, we recommend that hemodynamic data from First-In-Human studies be reported with all EOA, PDG, and MDG values for each valve size under a standardized timeframe. These parameters have been picked because they accurately describe the hemodynamic profile of the bioprosthesis. It should be recommended that early data is reported within 30 days, mid-term data reported at one year and 5-year mark, and long-term data at the 10-year mark. The relevant hemodynamic values in Additional file 1: Table S3 can be used as reference values for hemodynamic parameters when conducting the FIH.

### Morbidity and consequences

Only a handful of studies reported mortality (both early and late deaths) and structural valve deterioration; hence statistical comparison cannot be made between the subgroups. Older generation valves had a slightly higher percentage of early and late deaths than the newer generations, as shown in Additional file 1: Table S5. The older generation also has a much higher percentage of structural valve deterioration (11.4%) than the newer generations (0.0% and 1.2% in FIH and early clinical subgroups, respectively). FIH subgroup has lower mortality and structural valve deterioration than the early phase clinical trial subgroup, which can be explained by the fact that these adverse events take longer to develop, and hence would be present when there are more extended follow-up periods.

### Study protocol

**Fig. 5:**
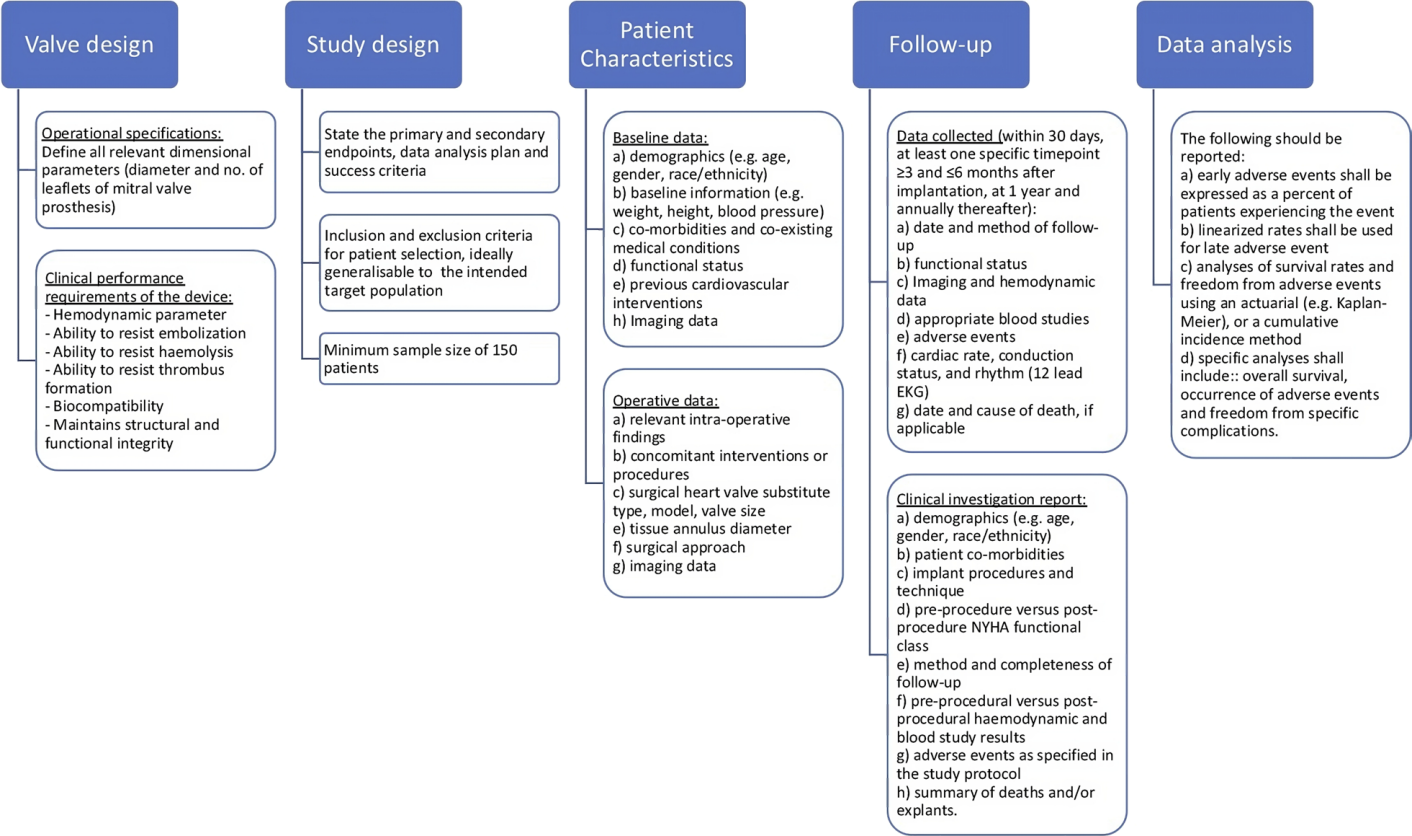
The clinical study protocol for a First-In-Human (FIH) trial of a surgically implantable mitral valve prosthesis is described in this summary. The process is broken down into five separate steps, and each stage encompasses critical factors to consider

The current review finds that heterogenicity in conducting FIH, or early clinical trial exists in terms of study characteristics, indication and timing of surgery, evaluation of new implantable surgical prosthesis, and the postoperative follow up for morbidity and consequences among the included studies [9–30]. Moreover, existing guidelines for managing valvular heart disease, as outlined by the ACC/AHA [33], do not offer clear directives tailored to FIH clinical trials. Similarly, available guidance materials for reporting mortality and morbidity after cardiac valve interventions were drafted over a decade ago [34] or predominantly focused on drug development [35]. There is currently no established framework specifically designed for FIH trials related to mitral valve surgeries. An intricate and crucial part of healthcare innovation is the problem of regulatory agencies and the approval procedure for new surgical implants. Rules and regulations, geographic inequalities, unmet medical needs, and the potential use of standardized templates for First-in-Human (FIH) clinical trials are just a few of the difficulties to name. Moreover, Considering the gaps and inconsistencies identified in the existing approaches, this review underscores the need for a standardized template for FIH clinical trial study protocols, particularly for mitral valve surgeries. As a response to these findings, we propose the development of such a template, as illustrated in Fig. 5, to provide a structured and comprehensive framework for conducting FIH trials in this context.

### Limitations

The scarcity of published studies incorporating FIH and early clinical trials has widened the range of inclusion criteria, exacerbating heterogeneity among research groups. Additionally, it is crucial to acknowledge the inconsistencies and imprecisions in the available research regarding the design and effectiveness of surgical mitral bioprostheses. Given these limitations and the limited reliable research, caution is warranted when drawing conclusions. Our utilization of the GRADE method for rating the quality of evidence allows for a more cautious and nuanced interpretation of the findings, considering the inherent constraints and uncertainties in the existing body of research.

## Conclusion

First-In-Human and early clinical trials are crucial steps for bio-prostheses development and help the transition to the application in human beings based on a streamlined process. Our study gives an overview of the history of the First-In-Human clinical trial for mitral bio-prostheses with a view to identify gaps in the current process. We also propose a First-In-Human clinical trial protocol to highlight the importance of standardising the clinical data to be collected during a “de novo” surgical implant whilst appreciating the challenges of innovation as essential lessons, which may offer insights into the future development of bio-prosthetic heart valves.

### Supplementary Information

**Additional file 1**. Supplementary tables that include risk of bias analysis of the included studies, prosthesis size, Hemodynamic data at 1 year, 2-9 years, and 10 years of follow-up, Test of Homogeneity of variances, Tests of equality of means (Welch), Morbidity and consequences in the included studies.

## Data Availability

The authors confirm that the data supporting the findings of this study are available within the article and its Additional file 1. Raw data were generated at the Cardiac Surgery Research Laboratory at the National University of Singapore. Derived data supporting the findings of this study are available from the corresponding author on request.
