# Supplementary material for: Systematic review of first-in-human and early phase clinical trials for surgically implantable biological mitral valve substitutes
Source: J Cardiothorac Surg. 2023 Nov 30;18:348. doi: 10.1186/s13019-023-02464-2 (PMC10688009; doi:10.1186/s13019-023-02464-2)
Supplement: Supplementary file 1 — Additional file 1. Supplementary tables that include risk of bias analysis of the included studies, prosthesis size, Hemodynamic data at 1 year, 2-9 years, and 10 years of follow-up, Test of Homogeneity of variances, Tests of equality of means (Welch), Morbidity and consequences in the included studies. [file 13019_2023_2464_MOESM1_ESM.docx]

**Supplementary Table 1:** Risk of Bias analysis by using GRADEpro

| **Study/Year** | **Risk of Bias** | **Inconsistency** | **Imprecision** | **Indirectness** | **Publication Bias** | **Certainty of the evidence (GRADE)** |
| --- | --- | --- | --- | --- | --- | --- |
| Relland 1985 | Not serious | Not serious | Not serious | Not serious | Undetected | ⨁⨁◯◯ LOW |
| Jamieson 1986 | Not serious | Not serious | Not serious | Not serious | Undetected | ⨁⨁◯◯ LOW |
| Jamieson 1998 | Not serious | Not serious | Not serious | Not serious | Undetected | ⨁⨁◯◯ LOW |
| Williams 1988 | Not serious | Not serious | Not serious | Not serious | Undetected | ⨁⨁◯◯ LOW |
| Loisance 1989 | Not serious | Not serious | Not serious | Not serious | Undetected | ⨁⨁◯◯ LOW |
| Bortolotti 1989 | Not serious | Not serious | Not serious | Not serious | Undetected | ⨁⨁◯◯ LOW |
| Garcia 1991 | Not serious | Not serious | Not serious | Not serious | Undetected | ⨁⨁◯◯ LOW |
| Wheatley 1995 | Not serious | Not serious | Not serious | Not serious | Undetected | ⨁⨁◯◯ LOW |
| Vermeulen 1992 | Not serious | Not serious | Not serious | Not serious | Undetected | ⨁⨁◯◯ LOW |
| Mohr 2005 | Not serious | Not serious | Not serious | Not serious | Undetected | ⨁⨁◯◯ LOW |
| Fradet 2001 | Not serious | Not serious | Not serious | Not serious | Undetected | ⨁⨁◯◯ LOW |
| Hiremath 2020 | Not serious | Not serious | Not serious | Not serious | Undetected | ⨁⨁◯◯ LOW |
| Firstenberg 2001 | Not serious | Not serious | Not serious | Not serious | Undetected | ⨁⨁◯◯ LOW |
| Folliguet 2001 | Not serious | Not serious | Not serious | Not serious | Undetected | ⨁⨁◯◯ LOW |
| Eichinger 2002 | Not serious | Not serious | Not serious | Not serious | Undetected | ⨁⨁◯◯ LOW |
| Masters 2004 | Not serious | Not serious | Not serious | Not serious | Undetected | ⨁⨁◯◯ LOW |
| Pomerantz 2006 | Not serious | Not serious | Not serious | Not serious | Undetected | ⨁⨁◯◯ LOW |
| Frater 2006 | Not serious | Not serious | Not serious | Not serious | Undetected | ⨁⨁◯◯ LOW |
| Riess 2007 | Not serious | Not serious | Not serious | Not serious | Undetected | ⨁⨁◯◯ LOW |
| Jamieson 2011 | Not serious | Not serious | Not serious | Not serious | Undetected | ⨁⨁◯◯ LOW |
| Loor 2016 | Not serious | Not serious | Not serious | Not serious | Undetected | ⨁⨁◯◯ LOW |
| Celiento 2016 | Not serious | Not serious | Not serious | Not serious | Undetected | ⨁⨁◯◯ LOW |

NB: Blue: Older FIM; Green: FIM; Orange: Early Clinical trials

**Supplementary Table 2: Prosthesis size**

| **Name/Year** | **Size (mm)** | **23** | **25** | **26** | **27** | **28** | **29** | **27 and 29** | **30** | **31** | **33** | **Total valve no** |
| --- | --- | --- | --- | --- | --- | --- | --- | --- | --- | --- | --- | --- |
| Williams 1988 | | - | 3 | - | 14 | - | 38 | - | - | 70 | 7 | 132 |
| Loisance 1989 | | 2 | - | - | - | - | - | 41 | - | 14 | - | 57 |
| Garcia 1991 | | - | - | - | - | - | - | 11 | - | - | - | 11 |
| Mohr 2005 | | - | - | 3 | - | 22 | - | - | 27 | - | - | 52 |
| Hiremath 2020 | | - | 5 | - | 12 | - | 3 | - | - | 10 | - | 30 |
| Firstenberg 2001 | | - | - | - | 14 | - | 26 | - | - | 31 | - | 71 |
| Folliguet 2001 | | - | - | - | 1 | - | 31 | - | - | 18 | - | 50 |
| Masters 2004 | | - | 9 | - | 25 | - | 43 | - | - | 22 | 1 | 100 |
| Riess 2007 | | - | mentioned | | | | | - | - | mentioned | | - |
| Jamieson 2011 | | - | 4.40% | - | 20.10% | - | 38.70% | - | - | 21.10% | 15.70% | 170 |
| Loor 2016 | | - | 18 | - | 16 | - | 19 | - | - | 16 | 1 | 70 |
| Celiento 2016 | | - | 3 | - | 50 | - | 40 | - | - | 7 | - | 100 |
| Total number across all studies | | 2 | 38 | 3 | 132 | 22 | 200 | 52 | 27 | 188 | 9 | 843 |

NB: Relland 1985, Jamieson 1986, Jamieson 1998, Bortolotti 1989, Vermeulen 1992, Fradet 2001, Eichinger 2002, Pomerantz 2006, Frater 2006: No data present; Wheatley 1995 presented the data in the form of bar charts that did not have any specific numbers.

**Supplementary Table 3: (A) Hemodynamic data at 1 year of follow up**

| **Name/Year** | **Prosthesis used** | **Valve Size** | **Reference** | **EOA (cm^2)** | **Overall** | **Reference** | **MDG (mmHg)** | **Overall** | **Reference** | **PDG (mmHg)** | **Overall** |
| --- | --- | --- | --- | --- | --- | --- | --- | --- | --- | --- | --- |
| Williams 1988 | Medtronic Intact (porcine) | 25 |  | 0.88 |  |  | 12 |  |  |  |  |
|  |  | 27 |  |  |  |  |  |  |  |  |  |
|  |  | 29 |  | 2.39 |  | 3.5 ± 0.51 | 5.3 |  |  |  |  |
|  |  | 31 |  | 1.92 |  | 4.2 ± 1.44 | 5.8 |  |  |  |  |
|  |  | 33 |  |  |  | 4 ± 1.3 |  |  |  |  |  |
|  |  | 35 |  |  |  | 3.2 ± 1.77 |  |  |  |  |  |
| Garcia 1991 | Labcor-Santiago (pericardial) | 25 | 2.2 |  |  | 4.5 |  |  | 8.7 |  |  |
|  |  | 27 | 2.12 ± 0.48 | 2.0 ± 0.1 |  | 2.8 ± 1.5 | 4.6 |  | 5.6 ± 2.3 | 8 |  |
|  |  | 29 | 2.11 ± 0.73 | 2.3 ± 0.1 |  | 3 ± 1.3 | 3.3 |  | 6.2 ± 2.1 | 7.3 |  |
|  |  | 31 |  |  |  |  |  |  |  |  |  |
|  |  | 33 |  |  |  |  |  |  |  |  |  |
| Wheatley 1995 | Bioflo (bovine pericardial) | 25 | 2 ± 0.1 |  |  | 6.3 ± 1.5 |  | 3.0 | 10 ± 2 |  |  |
|  |  | 27 | 2 ± 0.3 |  |  | 5.4 ± 1.2 |  |  | 9.5 ± 2.6 |  |  |
|  |  | 29 | 2.4 ± 0.2 |  |  | 3.6 ± 1 |  |  | 5 ± 2.8 |  |  |
|  |  | 31 | 2.3 |  |  | 2.0 |  |  | 4.0 |  |  |
|  |  | 33 |  |  |  |  |  |  |  |  |  |
| Mohr 2005 | Quattro* (stentless) | 25 |  |  | 2.6 ± 0.6 |  |  | 4.6 ± 1.4 |  |  |  |
|  |  | 27 |  |  |  |  |  |  |  |  |  |
|  |  | 29 |  |  |  |  |  |  |  |  |  |
|  |  | 31 |  |  |  |  |  |  |  |  |  |
|  |  | 33 |  |  |  |  |  |  |  |  |  |
| Fradet 2001 | Medtronic Mosaic (porcine)  Only aortic | 25 |  | 1.6 ± 0.4 |  |  | 5.7 ± 1.7 |  |  |  |  |
|  |  | 27 |  | 1.7 ± 0.5 |  |  | 4.6 ± 2.1 |  |  |  |  |
|  |  | 29 |  | 1.8 ± 0.5 |  |  | 4.4 ± 1.8 |  |  |  |  |
|  |  | 31 |  | 1.7 ± 0.5 |  |  | 3.7 ± 1.4 |  |  |  |  |
|  |  | 33 |  | 1.9 ± 0.5 |  |  | 3.4 ± 1.8 |  |  |  |  |
| Hiremath 2020 | Dafodil (stented, bovine, pericardial) | 25 |  |  | 2.67 ± 0.48 |  |  | 4.41 ± 1.69 |  |  |  |
|  |  | 27 |  |  |  |  |  |  |  |  |  |
|  |  | 29 |  |  |  |  |  |  |  |  |  |
|  |  | 31 |  |  |  |  |  |  |  |  |  |
| Eichinger 2002 | Medtronic Mosaic (porcine) | 25 |  | 1.8 ± 0.5 | 1.8 ± 0.6 |  | 6.5 ± 3.2 | 4.7 ± 1.8 |  |  |  |
|  |  | 27 |  | 1.9 ± 0.8 |  |  | 4.7 ± 1.7 |  |  |  |  |
|  |  | 29 |  | 1.9 ± 0.5 |  |  | 4.2 ± 1.6 |  |  |  |  |
|  |  | 31 |  | 1.6 ± 0.5 |  |  | 4.9 ± 1.3 |  |  |  |  |
|  |  | 33 |  | 2.5 |  |  | 6 |  |  |  |  |
| Riess 2007 | Medtronic Mosaic (porcine) | 25 |  | 1.9 ± 0.3 |  |  |  |  |  |  |  |
|  |  | 27 |  | 1.9 ± 0.4 |  |  |  |  |  |  |  |
|  |  | 29 |  | 2.0 ± 0.5 |  |  |  |  |  |  |  |
|  |  | 31 |  | 2.3 ± 0.6 |  |  |  |  |  |  |  |
|  |  | 33 |  | 1.9 ± 0.3 |  |  |  |  |  |  |  |

**Supplementary Table 3: (B) Hemodynamic data at 2-9 years of follow up**

| **Name/Year** | **Prosthesis used** | **Valve Size** | **Reference** | **EOA (cm^2)** | **Overall** | **Reference** | **MDG (mmHg)** | **Overall** | **Reference** | **PDG (mmHg)** | **Overall** |
| --- | --- | --- | --- | --- | --- | --- | --- | --- | --- | --- | --- |
| Wheatley 1995 | Bioflo (bovine pericardial) | 25 | 2 ± 0.1 |  |  | 6.3 ± 1.5 |  | 3.7 | 10 ± 2 |  |  |
|  |  | 27 | 2 ± 0.3 |  |  | 5.4 ± 1.2 |  |  | 9.5 ± 2.6 |  |  |
|  |  | 29 | 2.4 ± 0.2 |  |  | 3.6 ± 1 |  |  | 5 ± 2.8 |  |  |
|  |  | 31 | 2.3 |  |  | 2.0 |  |  | 4.0 |  |  |
|  |  | 33 |  |  |  |  |  |  |  |  |  |
| Mohr 2005 | Quattro (stentless) | 25 |  |  | 2.6 ± 0.3 |  |  | 4.9 ± 1.4 |  |  |  |
|  |  | 27 |  |  |  |  |  |  |  |  |  |
|  |  | 29 |  |  |  |  |  |  |  |  |  |
|  |  | 31 |  |  |  |  |  |  |  |  |  |
|  |  | 33 |  |  |  |  |  |  |  |  |  |
| Firstenberg 2001 | Carpentier-Edwards Perimount (pericardial) | 25 |  | 1.8 ± 0.4 | 2.5 ± 0.6 |  | 4.6 ± 1.9 | 4.4 ± 1.8 |  | 10.5 ± 2.4 | 9.1 ± 3.4 |
|  |  | 27 |  | 2.1 ± 0.8 |  | 3.6 | 4.5 ± 1.7 |  |  | 10.2 ± 3.3 |  |
|  |  | 29 |  | 2.5 ± 0.8 |  | 5.25 ± 2.36 | 4.5 ± 1.9 |  |  | 9.2 ± 3.0 |  |
|  |  | 31 |  | 2.5 ± 0.8 |  | 4.05 ± 0.83 | 4.2 ± 1.9 |  |  | 8.3 ± 4.0 |  |
|  |  | 33 |  | 2.3 ± 0.1 |  | 1.0 | 3.0 ± 1.9 |  |  | 7.1 ± 4.4 |  |
| Eichinger 2002 | Medtronic Mosaic (porcine) | 25 |  | 2.6 ± 0.6 | 1.9 ± 0.5 |  | 4.6 ± 1.9 | 4.1 ± 1.4 |  |  |  |
|  |  | 27 |  | 1.5 ± 0.3 |  |  | 3.8 ± 0.7 |  |  |  |  |
|  |  | 29 |  | 1.8 ± 0.6 |  |  | 4.4 ± 1.6 |  |  |  |  |
|  |  | 31 |  | 2.1 ± 0.3 |  |  | 2.7 ± 0.7 |  |  |  |  |
|  |  | 33 |  | 1.8 |  |  | 6 |  |  |  |  |
| Riess 2007 | Medtronic Mosaic (porcine) | 25 |  |  |  |  |  |  |  |  |  |
|  |  | 27 |  | 1.7 |  |  |  |  |  |  |  |
|  |  | 29 |  | 2.2 |  |  |  |  |  |  |  |
|  |  | 31 |  |  |  |  |  |  |  |  |  |
|  |  | 33 |  |  |  |  |  |  |  |  |  |
| Celiento 2016 | Medtronic Mosaic (porcine) | 25 |  |  |  |  |  |  |  |  |  |
|  |  | 27 |  | 1.57 ± 0.3 |  |  | 5 ± 1.7 |  |  | 12.6 ± 4 |  |
|  |  | 29 |  | 1.63 ± 0.4 |  |  | 4.5 ± 1.9 |  |  | 12.7 ± 4.7 |  |
|  |  | 31 |  |  |  |  |  |  |  |  |  |
|  |  | 33 |  |  |  |  |  |  |  |  |  |

**Supplementary Table 3: (C) Hemodynamic data at 10 years of follow up**

| **Name/Year** | **Prosthesis used** | **Valve Size** | **Reference** | **EOA (cm^2)** | **Overall** | **Reference** | **MDG (mmHg)** | **Overall** | **Reference** | **PDG (mmHg)** | **Overall** |
| --- | --- | --- | --- | --- | --- | --- | --- | --- | --- | --- | --- |
| Riess 2007 | Medtronic Mosaic (porcine) | 25 |  |  |  |  |  |  |  |  |  |
|  |  | 27 |  | 3.3 |  |  |  |  |  |  |  |
|  |  | 29 |  | 2.5 |  |  |  |  |  |  |  |
|  |  | 31 |  | 3.4 |  |  |  |  |  |  |  |
|  |  | 33 |  |  |  |  |  |  |  |  |  |

**Supplementary Table 4: (A) Test of Homogeneity of Variances**

|  | **Levene Statistic** | **df1** | **df2** | **Significance** |
| --- | --- | --- | --- | --- |
| Reoperation | 1.311 | 2 | 11 | 0.308 |
| Early Deaths | 6.536 | 2 | 16 | 0.008 |
| Late Deaths | 1.709 | 2 | 13 | 0.219 |
| SVD | 30.138 | 2 | 7 | 0.000 |
| Thromboembolism | 2.149 | 2 | 11 | 0.163 |
| Bleeding | 0.941 | 2 | 9 | 0.425 |
| Endocarditis | 1.149 | 2 | 9 | 0.359 |
| Periprosthetic Leak | 25.970 | 2 | 8 | 0.000 |

**Supplementary Table 4: (B) Tests of Equality of Means (Welch)**

|  | Statistic | df1 | df2 | Significance |
| --- | --- | --- | --- | --- |
| Early Deaths | 0.523 | 2 | 10.534 | 0.607 |
| SVD* | - | - | - | - |
| Periprosthetic Leak | 3.530 | 2 | 2.250 | 0.202 |

*Robust tests of equality of means cannot be performed for SVD because one group has 0 variance.

**Supplementary Table 5: Morbidity and consequences**

| **Subgroup** |  | **Early Deaths** | **Late Deaths** | **SVD** |
| --- | --- | --- | --- | --- |
| Older FIM | Mean | 6.8960 | 12.5025 | 11.4250 |
|  | N | 5 | 4 | 2 |
|  | Std. Deviation | 2.13244 | 13.51701 | 10.71267 |
| FIM | Mean | 5.4783 | 9.3760 | 0.00 |
|  | N | 6 | 5 | 3 |
|  | Std. Deviation | 3.28970 | 4.53630 | 0.00 |
| Early Clinical Trial | Mean | 5.3388 | 11.4486 | 1.2430 |
|  | N | 8 | 7 | 5 |
|  | Std. Deviation | 4.51736 | 9.78767 | 2.39481 |
